# The Acid–Base Flow Battery: Sustainable Energy Storage via Reversible Water Dissociation with Bipolar Membranes

**DOI:** 10.3390/membranes10120409

**Published:** 2020-12-10

**Authors:** Ragne Pärnamäe, Luigi Gurreri, Jan Post, Willem Johannes van Egmond, Andrea Culcasi, Michel Saakes, Jiajun Cen, Emil Goosen, Alessandro Tamburini, David A. Vermaas, Michele Tedesco

**Affiliations:** 1 Wetsus, European Centre of Excellence for Sustainable Water Technology, 8911 MA Leeuwarden, The Netherlands; ragne.parnamae@wetsus.nl (R.P.); jan.post@wetsus.nl (J.P.); michel.saakes@wetsus.nl (M.S.); 2 Dipartimento di Ingegneria, Università degli Studi di Palermo, viale delle Scienze Ed. 6, 90128 Palermo, Italy; andrea.culcasi@unipa.it (A.C.); alessandro.tamburini@unipa.it (A.T.); 3 AquaBattery B.V., Lijnbaan 3C, 2352 CK Leiderdorp, The Netherlands; janwillem.vanegmond@aquabattery.nl (W.J.v.E.); jiajun.cen@aquabattery.nl (J.C.); emil.goosen@aquabattery.nl (E.G.); david.vermaas@aquabattery.nl (D.A.V.); 4 Imperial College London, Department of Chemical Engineering, South Kensington Campus, London SW7 2AZ, UK; 5 Department of Chemical Engineering, Delft University of Technology, Van der Maasweg 9, 2629 HZ Delft, The Netherlands

**Keywords:** flow battery, energy storage, bipolar membrane, reverse electrodialysis, bipolar membrane electrodialysis, water dissociation

## Abstract

The increasing share of renewables in electric grids nowadays causes a growing daily and seasonal mismatch between electricity generation and demand. In this regard, novel energy storage systems need to be developed, to allow large-scale storage of the excess electricity during low-demand time, and its distribution during peak demand time. Acid–base flow battery (ABFB) is a novel and environmentally friendly technology based on the reversible water dissociation by bipolar membranes, and it stores electricity in the form of chemical energy in acid and base solutions. The technology has already been demonstrated at the laboratory scale, and the experimental testing of the first 1 kW pilot plant is currently ongoing. This work aims to describe the current development and the perspectives of the ABFB technology. In particular, we discuss the main technical challenges related to the development of battery components (membranes, electrolyte solutions, and stack design), as well as simulated scenarios, to demonstrate the technology at the kW–MW scale. Finally, we present an economic analysis for a first 100 kW commercial unit and suggest future directions for further technology scale-up and commercial deployment.

## 1. Introduction

The awareness of climate change and its alarming impact has resulted in the recognition of urgent need for decarbonization to stop further change. As coal-fired power plants alone account for almost a third of global CO_2_ emissions [1], the energy sector is under increased attention for its potential to remarkably reduce the emissions. To realize that potential, several climate mitigation strategies must be deployed at scale, such as carbon capture, utilization, and storage (CCUS), and increasing the share of nuclear power and renewables as energy source. However, the intermittent nature of renewables, such as solar and wind, presents a new challenge for electric grids, where the equality of power generation and consumption needs to be ensured with rapid adjustments. Hence, new technologies are needed to guarantee rapid adjustments and stabilization in modern grids with increasing share of renewables. In this regard, energy storage systems provide an excellent option for system stabilization. By storing energy while supply is larger than demand (and discharging energy back to the grid when the opposite occurs), energy storage systems can improve the flexibility and reliability of the grid. Moreover, since renewables often have distinct seasonal variations, there is especially a need for long-term (i.e., seasonal) energy storage.

**Figure 1:**
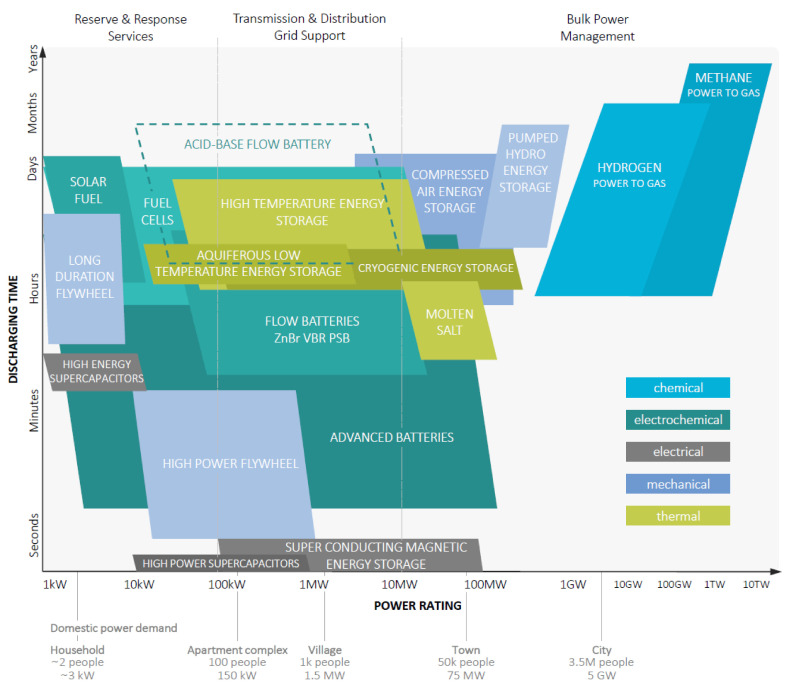
Energy storage systems and their conceptual comparison in terms of discharging time and power range. The figure is simplified, to give a qualitative comparison, and is not intended to be exhaustive; many of the storage systems can have broader operational ranges than shown. The domestic power demand scale is based on peak electric load demand of 3 kW per average EU household (~2 people). Adapted from Reference [6].

Although there are a number of technologies available for energy storage (Figure 1), only few of them are commercially deployed. Today, pumped hydro energy storage (PHS) is the most mature long-duration electricity storage system, and the only one commercially available at a large scale [1,2,3]. PHS systems store energy by moving water to a reservoir at elevated heights during times of low demand, and releasing it through a turbine into a lower reservoir during peak demand. PHS holds today the largest share among storage methods with over 120 GW installed electricity storage capacity for pure PHS plants (not receiving natural inflows, “closed-loop” plants) and almost 1.2 TW storage capacity for mixed PHS plants (both stored water and natural inflow used for generating electricity, “pump-back” plants) [4]. Due to its ability to store energy for up to months [5], and the emerging need to firm the seasonal fluctuations of renewables in grids, its installed capacity is anticipated to increase much more. However, PHS has major geographical constraints, as it needs large amounts of water, and elevated heights at site. Therefore, PHS is not suitable for flat and dry regions, as the construction of PHS plants would be clearly uneconomical in such locations. Suitable installation sites for PHS plants are mountainous regions with rivers (which are often protected natural areas). This raises some ecological and social concerns that need to be overcome when opting for PHS.

Electrochemical energy storage has received increasing attention as an alternative storage system [7,8,9,10,11], and several battery technologies have been making rapid advances in the past years. However, although batteries are commercial on a smaller scale, they are not yet widespread on larger scale nor in connection with electric grids. Larger-scale applications require specifically designed batteries. For example, lithium ion batteries are economically viable only for short-duration energy storage (<10 h discharge), where the value of the energy that they generate is higher than their own cost [12]; thus, they are unsuitable for the long-duration storage needed for renewables.

### Development, Principles, and State-of-the-Art of the Acid–Base Flow Battery

The acid–base flow battery (ABFB) can be considered as a modification of the concentration gradient flow battery [13], which relies on two opposite processes, i.e., electrodialysis (ED) and reverse electrodialysis (RED). Electrodialysis [14,15] exploits electric energy to desalinate a feed stream (typically brackish water). In conventional ED an electric field is applied over a membrane stack consisting of alternating anion- and cation-exchange membranes (AEMs and CEMs) that, by selective ion transport, separate the feed solution into a concentrate and a diluate stream, thus creating salinity gradients over the membranes. Over the past years, the opposite process, i.e., reverse electrodialysis (RED) [16,17], has also been widely investigated: In RED, two streams at a different salt concentration (i.e., concentrate and diluate) are fed to an analogous stack (with alternating AEMs and CEMs), so that the fluxes of cations and anions are driven by the concentration difference. As a result of the diffusive drive force inside the stack and the selective transport through the membranes, an ionic current can be harvested as electric current at the electrodes. Thus, in RED salinity gradients are used to produce electricity [18]. Interestingly, by coupling ED and RED processes in the same device, it is possible to create an energy storage system (known as ‘concentration gradient flow battery’, CGFB), to store electric energy in salinity gradients [19]. During the CGFB charging step (i.e., ED mode), electric energy is stored in generated salinity gradients. During the battery discharging step (RED mode), the previously generated salinity gradients are used to produce electricity. The CGFB technology has been demonstrated on laboratory scale [19] and on pilot scale [20]. A 1 kW/10 kWh pilot used to supply energy to a nearby student housing is operational since 2018 and located in Delft (The Netherlands).

In an ABFB, bipolar membranes are added auxiliary to CEMs and AEMs to generate a pH gradient in addition to the salinity gradient [21]. A bipolar membrane (BPM) is a composite membrane consisting of oppositely charged ion-exchange layers. In contrast to CEM or AEM that allow selective ion transport, the BPM (ideally) allows no transport of ions across it. Instead, the BPM is used to produce ions by dissociating water at the junction of its two layers [22,23]. Notably, unlike conventional water splitting occurring on electrode surfaces that produces gas (H_2_ and O_2_), the BPM-assisted water dissociation only generates ions (H^+^ and OH^−^), and occurs at a lower voltage (i.e., 0.83 V across a BPM separating 1 M HCl and 1 M NaOH instead of 1.23 V needed for water splitting in conventional electrolysis). No gas production inside the ABFB is also advantageous in terms of safety, compared to other battery systems based on electrolysis [24].

**Figure 2:**
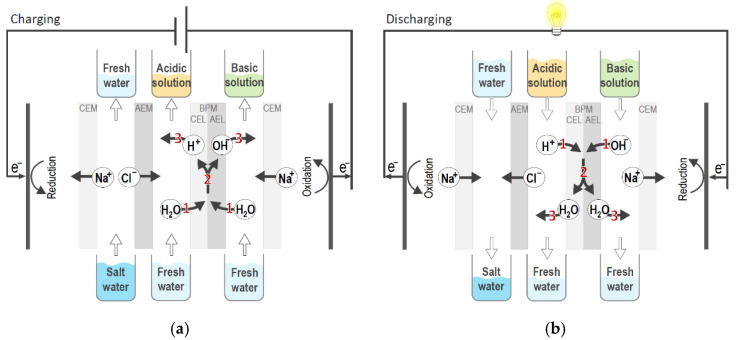
Schematic principle of the acid–base battery during (**a**) charging mode (bipolar membrane electrodialysis, BMED) and (**b**) discharging mode (bipolar membrane reverse electrodialysis, BMRED). Adapted from Reference [25].

The principle of ABFB is shown in Figure 2. During charging (Figure 2a), an electric field is applied over the stack and inside the BPMs water is dissociated into protons and hydroxyl ions. The produced ions leave the BPM junction through a respective BPM layer—protons through the cation-exchange layer (AEL), and hydroxide ions through the anion-exchange layer (AEL), meaning that H^+^ and OH^−^ ions leave the BPM on opposite sides. An additional salt (e.g., NaCl) is added to a third compartment in the repeating unit of the battery. As a result of the “salt ion” transport across monopolar membranes (i.e., Cl^−^ through AEMs and Na^+^ through CEMs), an acidic solution is obtained in one compartment (adjacent to the CEL of the BPM), and an alkaline solution in the other compartment (adjacent to the AEL of the BPM). Thus, two concentration gradients are produced over the BPM—(I) a pH gradient due to an acidic solution produced on one side, and an alkaline solution on the other side of the BPM, and (II) a salinity gradient due to the different composition of acidic and alkaline solutions. During battery discharge (Figure 2b), the electric field over the stack is opposite and the electric current flows through an external load, leading to the neutralization of acid and base solutions: H^+^ and OH^−^ ions flow into the BPM junction, where they recombine into water. Therefore, the ABFB charging step is bipolar membrane electrodialysis (BMED), and the discharging step bipolar membrane reverse electrodialysis (BMRED). Introducing BPMs (and, consequently, an additional pH gradient) in the battery increases the energy density of the battery significantly, i.e., by more than three times compared to the concentration gradient flow battery [25].

The ABFB technology is still at its early stage of development, with a very limited amount of works reported in the literature. The earliest study on this technology is from 1983 by Emrén and Holmström, who reported extremely low energy efficiency (0.1%), caused by poor permselectivity of the membranes and a high resistance of the (early stage) BPMs used at that time [26]. Pretz and Staude used the ABFB concept for a fuel cell application [27]. They operated the cell with acid–base concentrations up to 1 M HCl-NaOH, but observed irreversible water accumulation in the junction of the BPM, which led to delamination of the BPM. Zholkovskij et al. tested a similar battery to Emrén and Holmström (recirculating the salt solution while keeping acid and base compartments stagnant) [28]. They charged the battery only up to 0.03 M acid and base solutions, which also explains the low battery performance metrics (see Table 1). Kim et al. introduced Fe^2+^/Fe^3+^ redox couple in the electrode compartments of the ABFB to avoid electrolysis and subsequent gas formation at the electrodes [29]. However, because the electrode compartments were separated from the rest of the cell with CEMs, the battery performance suffered from iron ion migration from the electrode towards the base compartment, thus causing precipitation of iron salts. Van Egmond et al. demonstrated stable ABFB operation (at 150 A/m^2^ current density during charge, and 15 A/m^2^ during discharge) over a wide pH range (pH = 0–14), and analyzed the contribution of different energy loss sources [25]. They estimated the total energy lost by co-ion transport to be the biggest factor, contributing 39–65% of the total losses. Xia et al. investigated the ABFB on both single-cell [30] and stack (5–20 cell units) level [31], and concluded that the single cell performance can be extrapolated to the stack performance. However, additional energy losses by parasitic currents (also known as shortcut currents [32], shunt currents [31,33], or leakage currents [34]) through the manifolds need to be taken into account in the stack [31]. This aspect was highlighted by Culcasi et al., who modeled ABFB systems predicting a loss in round-trip efficiency in the range of 25–35% due to parasitic currents [35]. More recently, Zaffora et al. investigated the ABFB under different conditions of acid–base concentration (focusing on the discharge phase, similarly to Pretz and Staude [27]), and reported a maximum power density of 17 W/m^2^, and energy density of 10 kWh/m^3^ (1 M HCl-NaOH, at 100 A/m^2^ current density during discharge) [36].

The aim of this work is to describe the current development and technological challenges of the ABFB technology as a novel energy storage system. In particular, we focus on the main aspects related to the development of battery components (membranes, electrolyte solutions, stack design), and on modelled scenarios to demonstrate the technology at kW-scale. Finally, we present a preliminary techno-economic analysis of the technology, and suggest future direction for large-scale implementation.

## 2. Battery Components and Design

### 2.1. Bipolar and Monopolar Membranes

**Figure 3:**
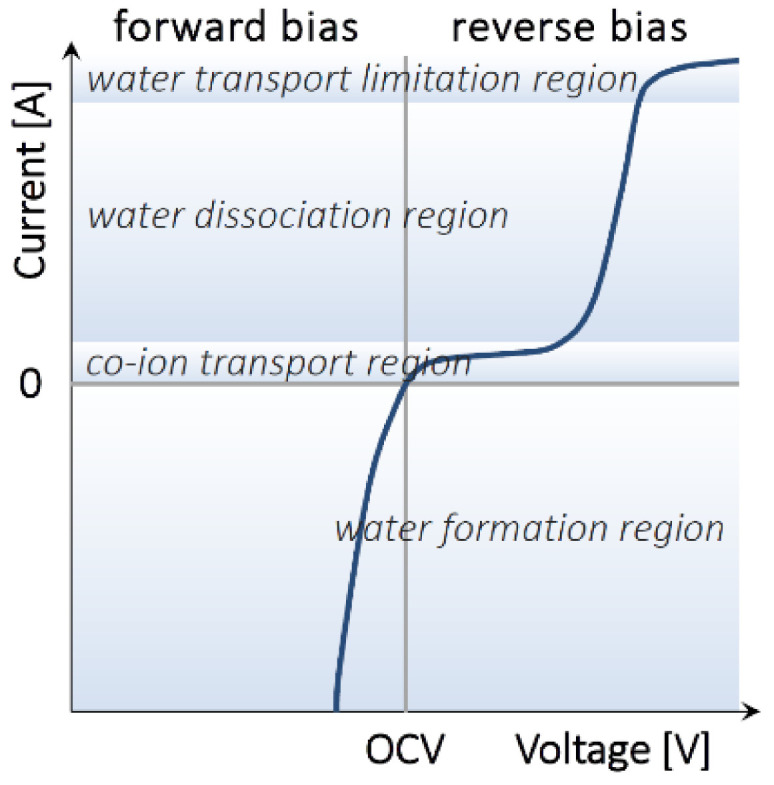
Typical current–voltage curve for a bipolar membrane. For the acid–base flow battery, forward bias corresponds to the battery discharge mode, and reverse bias to battery charge mode.

The core element of the ABFB is the bipolar membrane (BPM), which is responsible for the reversible water dissociation, and therefore, for the pH gradient in the battery. A BPM is an ion-exchange membrane consisting of two layers: a cation-exchange layer (CEL) and an anion-exchange layer (AEL). Contrary to conventional ion-exchange membranes (CEMs and AEMs), the function of a BPM is not to selectively transport ions from one side to the opposite one, as no ions can cross both layers of the membrane. In fact, ion transport across the BPM is unwanted. Instead, the function of a BPM is to dissociate water to protons and hydroxide ions at the junction (J) of its two layers (Figure 3).

If a high enough voltage is applied over the bipolar membrane, the water diffused into the membrane is dissociated into H^+^ and OH^−^ ions at the bipolar junction that carry the current demanded by the applied voltage. Commonly, a catalyst is introduced into the bipolar junction in order to promote water dissociation at lower voltage, thus lowering the energy requirements of the process [37,38,39,40,41].

The unique function of the bipolar membrane grants it some distinctive properties. While high water permeability is unwanted for ion-exchange membranes intended for separation processes (including the cation-and anion-exchange membranes in the ABFB which should have low water permeability to avoid diluting the acid and base compartments), it is a desired property for BPMs. When water is dissociated into ions at the BPM junction (battery charging mode, i.e., reverse bias in BPM literature), the junction must be constantly replenished with a diffusive water flux to ensure the membrane can withstand the current [42]. Oppositely, when H^+^ and OH^−^ ions are recombined at the junction (battery discharging mode, i.e., forward bias in BPM literature), the formed water must be able to diffuse out of the junction fast enough to avoid water accumulation at the junction and the consequent delamination of the bipolar membrane layers. Unlike all other bipolar membrane assisted processes [23], the acid–base flow battery uses the BPMs under both reverse bias (i.e., water dissociation) and forward bias (water recombination). Current commercial membranes are designed only for dissociating water, while operating BPMs under water recombination (which occurs during the discharging process in the ABFB), has been so far overlooked by membrane scientists and manufacturers. Hence, when using commercial BPMs in ABFB the discharge current densities of the battery are today limited by membrane delamination [25,30], and are thus relatively low. In particular, van Egmond et al. reported successful stable performance (up to 9 cycles) for a 1 M HCl-1 M NaOH battery operated at 15 A/m^2^ discharge current density [25], while Xia et al. noted water accumulation in the BPM junction when operating a 0.75 M HCl–0.75 M NaOH battery at discharge current densities above 200 A/m^2^ [30].

As with any electromembrane process, selectivity of the membranes is an important parameter also for the ABFB application. Both bipolar and the monopolar membranes should have high permselectivity to reduce co-ion leakage [43]. Co-ion leakage of “salt” ions (Na^+^, Cl^−^) causes the formation of neutral salt in the acid/base compartments, while co-ion leakage of water ions (H^+^ and OH^−^), i.e., the ion crossover through the entire BPM, causes water recombination in the outer solution (i.e., outside the BPM). In any case, the co-ion fluxes through (monopolar or bipolar) membranes lead to self-discharge of the battery. In addition, high selectivity of the monopolar membranes is crucial to avoid leakage of the electrode rinse solution into the acid and base compartments, as monopolar membranes are used for separating the electrode compartments from the rest of the cell, and the electrode rinse solution has a different composition from the rest of the battery [29]. Finally, due to the presence of highly acidic and alkaline solutions, all membranes in the ABFB need to be chemically stable in a wide pH range (pH = 0–14).

### 2.2. Battery Chemistry: Optimizing Electrolytes for Acid–Base Flow Batteries

The chemistry of acid–base flow batteries is based on the added electrolyte-the produced acid will consist of a proton from dissociation of water and the anion from the electrolyte, and the produced base of a hydroxide ion and the electrolyte cation. Thus, the choice of the electrolyte directly decides the composition of the acid and base produced for storing energy. In principle, any salt that is highly soluble in water, cheap, abundant, and that gives highly conductive solutions, could be potentially used as electrolyte in ABFBs. The main constraint for such salt is that it must be soluble not only in neutral conditions (aqueous solution), but also in acidic and alkaline conditions. In case of co-ion leakage into acid or base compartment an insoluble salt would otherwise precipitate and cause scaling on the membranes (CEM or BPM). For the same reason, the solubility requirement also applies for the acid and base produced from the salt, as well as for the electrode rinse solution. Thus, multivalent ions that lead to precipitation of hydroxides (e.g., Ca^2+^, Mg^2+^, and Fe^3+^), are not preferred as electrolytes in ABFBs [29,44,45,46].

The solubility limit of electrolytes in water is also directly connected to the battery storage capacity: A higher concentration of the acid and base solutions corresponds to a larger amount of energy stored in the battery. For example, considering the solubility limits of HCl and NaOH in water (12 and 19 M at 25 °C, respectively), the ABFB could theoretically be charged up to 12 M HCl-NaOH. Due to the solubility limit of NaCl in water being 6 M, the volume of NaCl solution should in such case be twice the volume of acid and base solutions in the battery. The Gibbs free energy for neutralization reaction of H^+^ with OH^−^ at room temperature is equal to the following:

(1) $$\Delta G=-R\cdot T\cdot \ln K_{\mathrm{eq}}=-1.99\cdot 10^{-3}\cdot 298.15\cdot \ln\frac{\left(1.0\cdot 10^{-14}\right)}{55.5}≅-25\;\mathrm{Wh}/{\mathrm{mol}}_{\mathrm{water}},$$

where *R* is the gas constant, T is temperature, and K_eq_ is the water dissociation constant. Accordingly, the theoretical storage capacity for 1 m^3^ of both HCl and NaOH solutions at 12 M concentration is ~300 kWh, which is remarkably high for a flow battery. However, uncontrolled mixing of such concentrated acid and base can be explosive, which raises new safety concerns when operating the battery. Furthermore, reaching such high acid–base concentrations in the battery is unpractical today, as for current commercial bipolar membranes 1 M acid–base concentration (theoretical energy density of ~25 kWh/m^3^) is the maximum practical value without sacrificing permselectivity [23]. Likewise, commercial monopolar membranes would suffer from severe co-ion leakage at such high concentrations. In other words, the bottleneck for increasing the ion concentration in ABFBs lies in the selectivity of monopolar/bipolar membranes. In particular, if new membranes with improved selectivity in highly concentrated solutions will be available, the ABFB capacity could be increased remarkably. Notably, even with a 2 M acid–base concentration (for a NaCl-HCl-NaOH system), the ABFB power density would be comparable with vanadium redox flow batteries [47]. Overall, the chemistry of the ABFB electrolyte is still largely unexplored, as all the reported studies so far focus only on the NaCl-HCl-NaOH system, with NaCl concentration in the range of 0.1 to 1 M (see Table 1).

### 2.3. Stack Design

The design of an acid–base flow battery resembles the typical design of a 3-compartment BMED stack [23], where a series of CEM, BPM, and AEM is used to create the repeating unit, or “triplet” (Figure 2). Each membrane is separated from its adjacent membranes with net spacers to create the compartments for salt, acid, and base solutions, and promote mixing. A gasket, either integrated with the spacer or not, is placed between two membranes to make the cell leak-proof. The gasket materials should be able to withstand highly concentrated acid and base solutions (i.e., using fluoroelastomers such as PVDF, FKM, FFKM, etc.). Long-term battery operation is possible only if the cell is acid and base resistant, has no internal leakages, and no extensive co-ion transport.

To increase power generation, multiple triplets are piled in one stack. An extra monopolar membrane is then added to close the membrane pile, and the whole series is placed between electrodes to form an ABFB stack. The electrode compartments can be rinsed with a different solution, for example Na_2_SO_4_ [30], to avoid the production of Cl_2_ at the anode (i.e., the oxidation product when using only NaCl solutions at the electrodes). The electrodes do not necessarily need to be of metal, but can also be of more environmentally friendly carbon material [25], in which case an electrode rinse solution with a redox couple must be used. Using such electrode rinse solution has the advantage of opposite electrode reactions occurring at the anode and cathode, meaning that by recirculating the rinse solution no net change of the chemical composition occurs, and the thermodynamic voltage of the electrode reactions is zero. In contrast to redox flow batteries, where electrodes or bipolar plates are needed between each repeating unit, a single ABFB stack contains only two end electrodes.

The feed flow in the triplets of the stack can be either parallel or serial. In case of parallel flow, all the compartments are simultaneously fed directly from the external electrolyte solution vessels. In case of serial flow, the feed solutions from the external vessel are fed into the first cell unit of the stack and the next cell unit receives the solution from the previous cell unit. At a given total flow rate, parallel flow has the advantage of lower pressure drop compared to serial flow, where the solutions flow through the entire stack. However, parallel flow has less homogenous flow as the compartment resistances throughout the stack might vary. In addition, it causes higher parasitic current losses, which has been described by Xia et al. [31,48]. When an external voltage is applied over the stack, protons and hydroxide ions migrate through the manifolds in opposite direction from one side of the stack to the other. This means that at the center of the stack the sum of their parasitic ion fluxes is the highest. The parasitic H^+^ and OH^−^ ion fluxes, and the compensating H^+^ and OH^−^ ion fluxes in the opposite direction at the stack center lead to water recombination inside the central BPMs. In practice, this results in self-discharge of the battery. As such, self-discharge phenomenon is limited only to the stack and does not include the feed storage vessels, reducing the manifolds size (by instance reducing the diameter) is necessary to decrease the effect of parasitic currents and self-discharge of the battery. In addition, the stack should have an optimal number of cell units to reduce the manifolds length and therefore the risk of self-discharging. Thus, instead of stacking together hundreds of cells, it can be more practical to connect together multiple stacks. These stacks should be hydraulically connected in parallel, so all stacks would work at the same concentration gradient, but electrically in series (to avoid reverse polarity instances between stacks). Another option to reduce parasitic currents is using independent hydraulic circuits for small blocks of triplets.

The ABFB energy and power ratings are independent of each other, as is the case with any flow battery. In practice, this means that the volume and concentration of the electrolyte solutions define the storage capacity of the battery, while the active area of the stack determines its power rating. This is a clear advantage for the ABFB in terms of scalability, especially when using an abundant and cheap salt (such as NaCl) as electrolyte. Moreover, the energy cost per kWh decreases with increasing battery capacity [49].

## 3. Simulation of Upscaled Scenarios for Technology Demonstration at kW–MW Scale

To evaluate the feasibility of the ABFB technology at larger scale, we performed a sensitivity analysis with a large-scale multi-stage (battery stacks hydraulically in series) ABFB under different scenarios, especially focusing on the effect of the number of battery stacks in series. The ABFB was simulated by using the process model previously developed and validated against laboratory experimental data by Culcasi et al. [35]. This model requires electrochemical and transport properties of the membranes as input parameters, and can predict the behavior and performance of the ABFB for different geometrical configurations and operating conditions as output.

The modelling tool has distributed parameters and is based on a hierarchical simulation strategy (multi-scale approach [35]), which can be briefly described as follows. The lowest level of simulation is represented by a single channel, where the model computes the physical properties of the electrolyte solutions, and computational fluid dynamics (CFD) correlations are used to calculate concentration polarization phenomena and pressure losses. The middle-low level is given by the “triplet” model (i.e., the repeating unit of the ABFB), which describes the mass balance and transport of water and ions (Nernst–Planck–Donnan approach) through both monopolar and bipolar membranes. Moreover, the “triplet” model evaluates also electrical variables as the resistance of the repetitive unit and the electromotive force (Nernst equation for multi-electrolyte solutions [50]). These first two modelling levels present a computational domain discretized along the channel length (where 30 intervals were sufficient to reach numerical accuracy at the simulated battery size). The middle-high level simulates the hydraulic and the electrical behavior of the stack. Note that the design features adopted in the present simulations were purposely chosen to minimize pressure drops and parasitic currents (<~5% of the gross power). Finally, the highest-scale model is able to simulate the external hydraulic circuit, including the dynamic mass balance in all the vessels, as well as pressure drops in the external piping. In the present work, we have assumed only once-through operations with negligible pressure drops in the external circuit. For the sake of brevity, only the main modeling results and the definition of output variables are reported in this work, while a more detailed mathematical description of the model can be found in Reference [35].

The sensitivity analysis was performed by using the input parameters shown in Table 2. In particular, different multi-stage operations were simulated by varying the number of battery stacks from 4 up to 17 (i.e., batteries hydraulically connected in series, fed with single pass through all 4 to 17 stacks, or “stages”). In all simulations, we assumed a battery state of charge (SOC) of 0% at 0.05 M and 100% at 1.00 M HCl, thus fixing the concentration targets at the outlet of the last stage for both charge and discharge. The electric current was tuned accordingly to achieve the concentration targets in all the simulated scenarios. For the sake of simplicity, the same electric current was used in all the sequential stages. Steady-state simulations were performed, assuming a single charge–discharge cycle.

The following model outputs are defined to characterize the battery performance. The gross power density (GPD, in watt per m^2^ of total membrane area) of the *k*-th stage is calculated as

(2) $$GPD_{k}=\frac{I_{ext}U_{ext,k}}{3\;N\;b\;L},$$

where $I_{ext}$ is the electric current in the external circuit (note that $I_{ext}$ changes with the number of stages, but is the same for all sequential stages), $U_{ext,k}$ is the predicted voltage on the external load (during discharge phase) or the power supply (charge phase), N is the number of repeating units (i.e., 10 triplets) of each stack, and b and L are the width and length of the membrane active area. Note that the power density in Equation (2) is normalized for the total membrane area, i.e., taking into account three membranes (CEM, AEM, and BPM) for each triplet, and it must be multiplied by a factor 3, to obtain a power density per square meter of BPM or triplet (as used in other works in the literature). The power output of the *k*-th stack ($P_{k}$) is equal to $P_{k}=I_{ext}U_{ext,k}$. The resulting gross power density of the multi-stage ABFB system is given by the average GPD over all the $N_{s}$ sequential stages:

(3) $$\bar{GPD}=\frac{\sum_{k=1}^{N_{s}}GPD_{k}}{N_{s}}.$$

The gross power (*P*) of an ABFB plant of generic size (i.e., of a given $N_{p}$ number of stacks hydraulically in parallel) is equal to the following:

(4) $$P=N_{p}\sum_{k=1}^{N_{s}}P_{k}.$$

The discharge gross energy density ($GED_{d}$) is calculated as follows:

(5) $$GED_{d}=\frac{\sum_{k=1}^{N_{s}}P_{k,d}}{Q_{a}},$$

in which $P_{k,d}$ is the discharge power of the *k*-th stage and $Q_{a}$ is the volumetric flow rate of acid solution. The discharge energy efficiency ($\eta_{d}$) is calculated as follows:

(6) $$\eta_{d}=\frac{GED_{d}}{GED_{th,d}},$$

in which $GED_{th,d}$ is the theoretical energy density, equal to 24 kWh/m^3^ (normalized on the volume of one feed solution) at 1 M HCl-NaOH [25]. Finally, the main figure of merits characterizing the charge–discharge cycle of the ABFB are the coulombic efficiency (CE), voltage efficiency (VE), and round-trip efficiency (RTE), defined as follows:

(7) $$CE=\frac{I_{ext,d}}{I_{ext,c}},$$

(8) $$VE=\frac{\bar{U_{ext,d}}}{\bar{U_{ext,c}}},$$

(9) $$RTE=\frac{I_{ext,d}\times \bar{U_{ext,d}}}{I_{ext,c}\times \bar{U_{ext,c}}}=CE\times VE,$$

in which, $\bar{U_{ext,d}}$ and $\bar{U_{ext,c}}$ are the average values of external voltage during discharge and charge, respectively, and $I_{ext,d}$ and $I_{ext,c}$ are the corresponding external currents (equal for all stages).

For comparison, we have also simulated the ABFB pilot plant as described in Section 4 (i.e., four hydraulically parallel stacks, with 56 triplets per stack). The main stack features and model input correspond to those reported in Table 2. In addition, each stack of the pilot plant is divided into eight blocks with independent hydraulic circuit. Accordingly, one seven-triplet block was simulated with an additional resistance of 6.5 Ω cm^2^ attributed to the supplementary components used for dividing a stack into multiple blocks. The ABFB pilot was simulated as a single stage system operating in dynamic mode with the solutions continuously recirculated in the tanks, which were assumed with perfect mixing. The volumes of solutions per block were considered 62.5 L for the acid and base solutions, and 312.5 L for the salt solution (i.e., 500 L of acid and base solutions per stack, and 2500 L of salt solution per stack). All the performance parameters were calculated as time averages with corresponding changes with respect to the above definitions. Both charge and discharge phases were simulated with fixed current density of 100 A/m^2^. The CE was calculated as the ratio between the total charge transferred in the discharge phase and that in the charge phase.

### 3.1. Performance of Upscaled Multi-Stage ABFB System as Function of the Number of Stages

**Figure 4:**
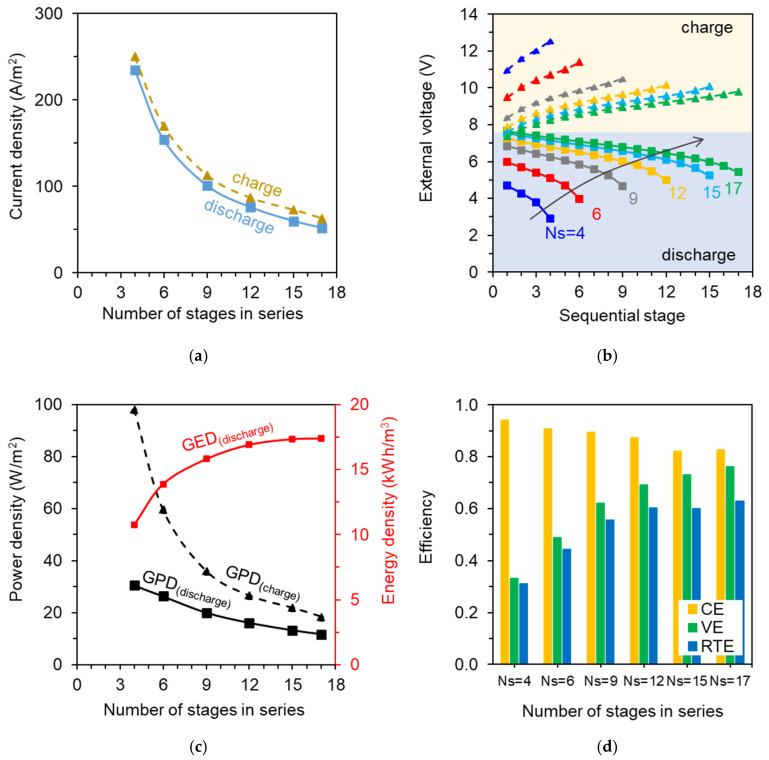
Model predictions of the effect of number of stages in series for an acid–base flow battery system: (**a**) charge and discharge current density (fixed equal for all stages); (**b**) profiles of charge/discharge external voltage at each sequential ABFB stage; (**c**) average charge/discharge gross power density (*GPD*) and (discharge) gross energy density (*GED*); (**d**) Coulombic efficiency (*CE*), voltage efficiency (*VE*), and round-trip efficiency (*RTE*). Each stage is simulated as an ABFB stack with a membrane active area of 0.5 × 0.5 m^2^.

The main simulation results of the multi-stage ABFB are reported in Figure 4, as well as the applied values of current density, highlighting the effect of the number of stages on the process performance. In particular, Figure 4a shows the current density (i.e., current divided by the membrane active area) that was required to achieve the same (inlet–outlet) concentration difference for the acid solution, as a function of the number of ABFB stages. As expected, the required current density decreases as the number of sequential stages *N_s_* increases, due to the accompanying increase in total membrane area (at fixed feed flow rate). However, the total electric current is not constant. As *N_s_* increases, the total electric current increases from ~250 to ~270 A in charge, and it decreases from ~235 to ~221 A in discharge, thus indicating a decreasing current efficiency in both phases. This is simply caused by the increasing total membrane area, and consequently, increasing total mass transported by undesired fluxes of co-ions and water.

The predicted values of voltage over all stacks are reported in Figure 4b. During the discharge phase, the external voltage decreases along the stages due to the decreasing driving force (i.e., pH and concentration difference). Likewise, the voltage increases along the stages during charge, as a result of the increasing concentration difference. As the number of stages in series increases (Figure 4b), the voltage profiles along the stages tend towards the open circuit conditions (both for discharge and charge), as a result of the decreasing current density in the stacks (Figure 4a). The average gross power density ($\bar{GPD}$) of the stacks series (Figure 4c) exhibits a decreasing trend with the number of stages (*N_s_*), similarly to the electric current. During the charge phase both the electric current and the average voltage decreases with *N_s_*, thus causing a more pronounced reduction of $\bar{GPD}$ than during the discharge phase. The predicted $\bar{GPD}$ values are in the range of 18.5–98.2 W/m^2^ for charge, and 11.7–30.6 W/m^2^ for discharge.

The discharge gross energy density ($GED_{d}$) is reported also in Figure 4c. The gross energy density is equal to 10.4 kWh/m^3^_acid_ for the four-stage system (discharge energy efficiency $\eta_{d}$ of ~45%), and it increases with the number of stages due to the increasing cumulative power, reaching a plateau at $GED_{d}$ = 17.4 kWh/m^3^_acid_ for the 17-stage system ($\eta_{d}$ ≈ 72%). This indicates that the overall power ($\overset{N_{s}}{\underset{k=1}{\sum }}P_{k,d}$) increased at decreasing rate as a function of *N_s_*, eventually reaching a maximum value. This is because the simulations at higher numbers of stages were based on lower current density values, thus distant from peak power conditions in discharge. The discharge electrical efficiency, defined as the power delivered to the external load divided by the total dissipated power (i.e., the sum of the internal and external power) was comprised between 47% (*N_s_* = 4) and 79% (*N_s_* = 17). Moreover, the less than proportional increase of the overall power with the number of stages justifies the reduction of $\bar{GPD}$ Figure 4d shows the efficiency of the process, in terms of coulombic efficiency (*CE*), voltage efficiency (*VE*), and round-trip efficiency (*RTE*). The *CE* decreases from 94% to 82% as *N_s_* increases. Such high values of coulombic efficiency mean that the battery is characterized by high current efficiencies in both phases (charge/discharge). In particular, since parasitic currents in the manifolds are negligible in this case (due to small manifolds size and low number of triplets), the current efficiency is affected only by undesired fluxes (of co-ions and water) through the membranes. The voltage efficiency (*VE*) increases with the number of stages (from 33% up to 76%), as a result of the more homogeneous voltage distribution among sequential stages both during charge and discharge (Figure 4b). As overall result, the round-trip efficiency (*RTE*) trend is essentially determined by the increase of the *VE*, with *RTE* values in the range of 31–63% by increasing the number of stages.

The results of the pilot plant simulation are summarized in Table 3. The multi-stage ABFB and the pilot plant have different features, and thus a direct comparison of their performance parameters is not possible. However, it can be observed that the pilot plant (four stacks in parallel) performance is similar to the performance of a nine-stage ABFB (nine stacks in series), where similar values of current density (i.e., ~100 A/m^2^) were applied.

**Figure 5:**
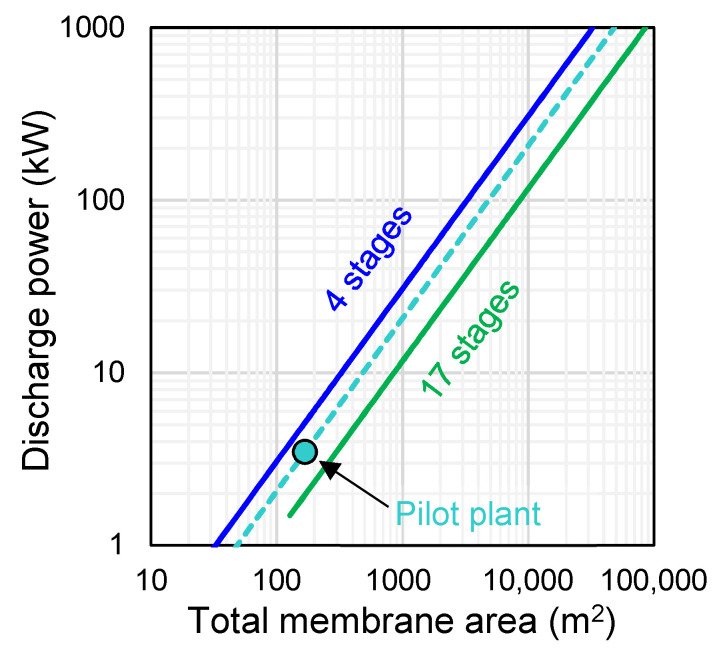
Discharge power as a function of the total membrane area for four-stage (*N_s_* = 4) and 17-stage (*N_s_* = 17) ABFB systems, and for the single stage pilot system (the highlighted point refers to the four-stack pilot plant). Each stage/stack is simulated with a membrane active area of 0.5 × 0.5 m^2^.

Figure 5 shows the predicted output power (Equation (4)) as a function of the total membrane area (increased by increasing the number of parallel stages) for the cases of *N_s_* = 4 and *N_s_* = 17 sequential stages. With the current performance of commercial (monopolar/bipolar) membranes, a discharge power of 1 kW could be achieved by using a four-stage ABFB system with a total membrane area of about 30 m^2^ (i.e., each stage equipped with 10 triplets and a membrane active area of 0.5 × 0.5 m^2^). For a 1 MW power system, the required membrane area increased up to 30,000 m^2^ (i.e., ~10,000 parallel triplets for each stage of the four serial stages). For the case of 17 stacks in series, the membrane area providing the same discharge power is increased by 2.6 times (6730 parallel triplets for 1 MW). Given the energy density of the system (Figure 4c), the corresponding flow rates to supply 1 MW power are 93 and 58 m^3^/h (for each solution) for the four-stage and 17-stage ABFB systems, respectively. These modelling results highlight that, to reach ABFB applications on the kW–MW scale, further optimization studies should focus on plant design to reduce the membrane area and volume of solutions. The pilot plant system is also represented in Figure 5. With a fixed current density of 100 A/m^2^, the plant consisting of four stacks with eight blocks of seven triplets each (total membrane area of 168 m^2^) could provide a power output of 3.46 kW. Compared to the four-stage system, it requires ~60% more membrane area to deliver the same power.

## 4. Techno-Economic Assessment of First Pilot Plant and Technology Scale-Up

The development of ABFB technology is rapidly growing, and in 2020, a first pilot-scale demonstration plant with a target capacity of 1 kW/7 kWh was constructed by AquaBattery B.V. The pilot was recently installed in Pantelleria (a small Italian island in the Mediterranean Sea) and will be tested in the upcoming months as energy storage system at the local power plant, to provide seasonal storage during the high energy demand in summer months. In this section, we give a cost breakdown of the construction of this pilot plant, and we estimate a cost projection for a future 100× upscaled plant (i.e., a 100 kW/700 kWh full-scale unit), taking into account technology development and estimated prices in the next five years (2021–2025). Finally, we compare the cost of the ABFB with redox flow batteries.

The ABFB system, and hence the related costs, can be divided into three main subsystems: (i) a power subsystem, comprising all components related to the stack and the battery triplets (determining the power rating); (ii) an energy subsystem, comprising the volume of electrolyte solutions and associated components (determining the storage capacity); and (iii) the periphery, including all the auxiliary components that are not scale-dependent (e.g., battery management system). Location-dependent components needed for the battery integration into an existing built environment are not taken into consideration in this cost analysis.

The pilot plant consists of a four-stack (hydraulically parallel) ABFB system, where each stack contains 56 triplets with a membrane active area of 0.5 × 0.5 m^2^ and spacer thickness of 475 μm, with a co-flow arrangement inside each stack. Therefore, each ABFB stack is designed to deliver a power output of 250 W, with an average power density of 6 W/m^2^ membrane. Such low power density is due to the discharge current density of the pilot plant being limited to 30 A/m^2^ during the preliminary testing phase for avoiding any risks of BPM delamination, and thus operating the stack far from the peak power condition (maximum of GPD-i curve). However, the plant is planned to operate closer to peak power at a later testing phase. The cost breakdown is summarized in Table 4. The total cost of membranes and spacers of this pilot plant was €63,000, which accounts for 74% of the total cost (€85,000) of the power unit. It should be noted that membrane and spacer costs are rather high (~€1000/m^2^ of triplet), as the costs are related to a pilot-scale project, and are therefore affected by large R&D costs (e.g., due to the relatively small quantity of tailored non-commercial membranes that had to be produced). The energy storage capacity of the pilot (7 kWh), consisting of water storage tanks for the acid and base (2000 L each), salt (10,000 L tank for 4000 L solution), and electrode rinse solutions (25 L), cost €13,000. The periphery, containing the electrical cabinet and sensors among others, cost €39,000. Table 4 also shows the cost estimation for a First-of-a-Kind (FOAK) commercial unit.

To estimate the costs for a 100 kW FOAK commercial unit, we consider a four-stack ABFB with each stack delivering a peak power output of 25 kW. This corresponds to upscaling the current demonstration pilot by a factor of 16 for the total membrane area and could be achieved by deploying four stacks with 224 triplets and membrane active area of 0.5 × 2.0 m^2^. By upscaling the technology, the production cost of membranes and spacers will further decrease, leading to expected membrane/spacer costs in the range of €100/m^2^ per triplet (i.e., for three membranes and spacers). Moreover, the development of bipolar membranes specifically tailored for the ABFB application (i.e., able to withstand high current densities under forward bias without delaminating) will allow to operate the ABFB at higher discharge current densities, and hence to increase the power density. Power density of 17 W/m^2^ total membrane area has already been achieved at the lab scale [36], and simulations by our model predict power densities up to 30 W/m^2^ (Section 3.1). Taking into consideration that the development of new membranes with lower resistance will decrease the internal resistance of the battery stack in the future, power densities in the range of 30–40 W/m^2^ (100 W/m^2^ triplet assumed for the cost calculations) could be realistically achieved. Accordingly, the power subsystem (only membranes) costs for the 100 kW commercial unit result in a total of €152,000 (Table 4). The substantial reduction in price compared to the pilot is a combination of the aforementioned factors: reduced membrane price, due to economies of scale and ongoing development of membrane manufacturing, and improved power density (6 to 30–40 W/m^2^ in future). The costs of the energy subsystem (€35,000) include the use of low-cost water storage bags instead of water tanks for the feed solutions. The periphery costs account for additional €22,000.

Interestingly, according to Table 4, the power subsystem costs and energy subsystem costs for the FOAK commercial unit are €1520/kW and €50/kWh, respectively. These power unit costs are comparable with large-scale vanadium redox flow batteries mentioned in literature, i.e., in the range of ~€1000/kW for the power subsystem [51,52]. The energy unit costs are, however, significantly lower than for the competing vanadium-based flow battery technologies, which are in the range of €250–400/kWh [51,52,53]. Compared to other flow batteries, the ABFB is especially attractive for long-term storage, due to the relatively low cost of the energy subsystem.

**Figure 6:**
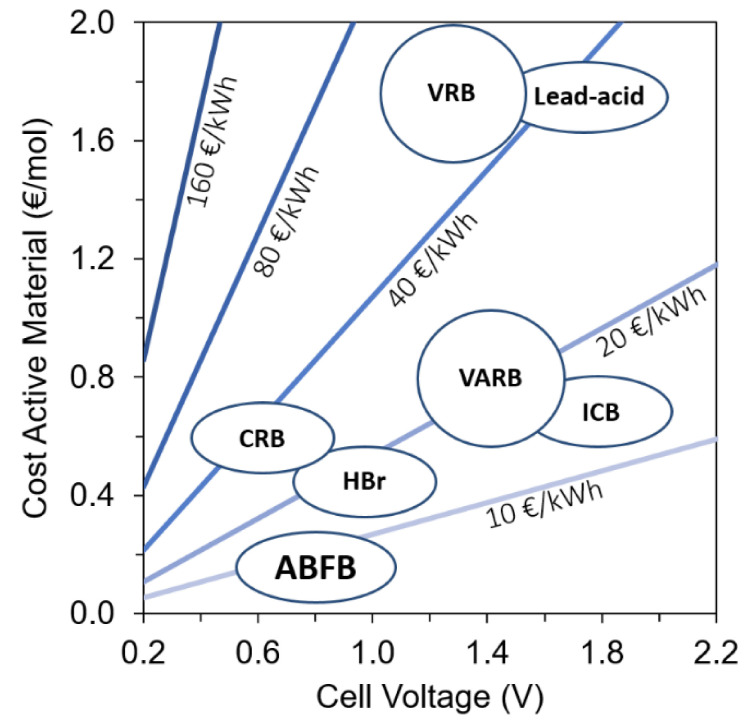
Energy costs (€/kWh) related to the active materials used for storage in the ABFB and various redox flow batteries. CRB, all-copper redox flow battery; HBr, hydrogen bromine redox flow battery; ICB, iron chromium redox flow battery; VARB, vanadium/air redox flow battery; VRB, vanadium redox flow battery.

Since the cost levels are dependent on both material and technology development, a crucial aspect that determines the costs of flow batteries is the cost of the active materials involved in the storage capacity (i.e., the redox couples in the case of redox flow batteries). Figure 6 presents an overview of the cost of active materials for several redox flow battery (RFB) technologies and the ABFB, and it shows that the resulting energy storage costs related to the storage medium for ABFB are far lower than for other flow batteries. In other words, since energy is stored in (abundantly available) salt solutions, the ABFB has the potential to be a truly sustainable and cost-effective battery technology for stationary energy storage at a large scale.

## 5. Outlook and Perspectives

Acid–base flow batteries represent a promising technology to provide safe and sustainable storage in many applications. While the energy density of ABFB is comparable with PHS [25], it is relatively low compared to other batteries, and thus the potential applications of ABFBs are on a somewhat smaller scale (Figure 1). This, however, can be an advantage, as ABFBs can be brought closer to the consumer: Distributed batteries at the household level (i.e., “behind-the-meter” batteries) offer a larger amount of services and thus contribute to the electrical system the most. While an “in-front-of-the-meter” battery supports the grid, a behind-the-meter battery can additionally be used for customer services (e.g., for managing electric bills or, more importantly, for backup power). Although the ABFB technology is also suitable as an in-front-of-the-meter battery for secluded estates in regions with weak electric grids (for example small islands) and for solar or wind farms, its main application could be as a behind-the-meter battery for neighboring house groups or apartment complexes in areas with large share of renewables. The non-toxic and safe battery chemistry (which is based on NaCl and water) further justifies the suitability of ABFBs for household level, by eliminating many safety concerns relevant to other battery systems. In addition, as the battery chemistry is based on abundant salts, the costs for the active material of the battery are exceptionally low. To reduce the costs even further, ABFBs could in principle operate with natural saline waters (e.g., brackish water and seawater) or industrial waste waters as electrolyte solutions (though the use of natural feed waters would require additional pretreatment costs to avoid membrane scaling and fouling). Using industrial waste streams for energy storage via ABFBs can additionally reduce brine disposal costs and contribute to the development of zero liquid discharge processes in the future.

One of the main technical challenges of the ABFB technology is to improve the BPM performance, especially to increase its stability and selectivity under forward bias (i.e., water formation) conditions, which would allow for fast discharge of the battery. In fact, fast discharge of the ABFB is today unfeasible due to the limited performance of the available bipolar membranes; however, this is expected to change in the near future. The rapidly increasing attention towards BPMs over the last two decades has already led to major improvements in the BPM properties [23]. For instance, electrospun bipolar membranes with 3D junction have shown to be stable at unprecedentedly high values of current density [54], and could be therefore suitable for ABFB applications. A new class of BPMs might emerge in the future, with focus on high performance during both forward and reverse bias, and therefore with optimized properties for energy storage applications. This might lead to new insights also on the composition of the BPM junction, as there is no evidence that a good catalyst for water dissociation (i.e., reverse bias) can also catalyze the opposite process (water formation), since the behavior of BPMs under forward bias is essentially unexplored in the literature. Ultimately, bipolar membranes have become a quickly expanding market in recent years, with several new manufacturers (e.g., Xergy [55], Weifang Senya Chemical [56], and others [23]) on the market. Thus, it is justified to expect that the upscaling of ABFB technology (which has the bipolar membrane as its core element) will soon benefit from the R&D advances in the field.

Improvements in the properties of monopolar membranes will also benefit the process. Achieving low resistance, co-ion leakage, and water permeability at the same time is very challenging. However, research activities on the development of novel high-performing membranes are currently intense and promising. Moreover, the optimization of stack design and operating conditions will be crucial for the process competitiveness. In this regard, several flow layouts can be adopted, e.g., single or multi-stage/stack, batch (recirculation) or sequential [57]. Efforts on the component development and process optimization will lead to better performances, e.g., higher values of power density, RTE, and number of cycles. Finally, the abatement of the membrane cost is crucial for the techno-economic feasibility of ABFB systems and their actual implementation at a large scale.

## 6. Conclusions

The aim of this work is to present the state-of-the-art and latest developments of acid–base flow batteries (ABFBs) as a promising technology to provide seasonal energy storage by means of water dissociation with bipolar membranes. While still at the early stage of development, the ABFB technology is gaining attention and has been recently demonstrated at the pilot scale, for seasonal storage. To evaluate the feasibility of the ABFB technology at a larger scale, different scenarios of multi-stage (from 4 to 17) operation were simulated by fixing the same concentration target at the last stage. The results showed average values of discharge power density decreasing from 30.6 to 11.7 W/m^2^ membrane, while the energy density increased from 10.7 to 17.4 kWh/m^3^ acid. This means a total membrane area of ~30 to 86 m^2^/kW discharge power and a volume of each electrolyte solution of 0.09–0.06 m^3^/kWh. The round-trip efficiency increased from 31% to 63%. Improved membranes and optimized systems can lead to enhanced performances, thus reducing the electrolyte volumes and the membrane area. The simulation of the pilot plant showed results in line with the multi-stage systems, being potentially able to deliver a discharge power density of ~21 W/m^2^ total membrane area.

As the performance of the ABFB is tightly connected to its core component, expected improvements of bipolar membranes in the near future will also directly improve the battery. In particular, tailored bipolar membranes that are able to withstand high current densities under forward bias (battery discharge mode) are needed to enable fast discharge of the ABFB. Despite all of the significant advancements on several battery technologies during the past decade, there is still a need for novel and sustainable energy storage systems for long-duration storage. In this regard, thanks to the safe and cost-effective battery chemistry, the acid–base flow battery can play a role towards the development of environmentally safe and sustainable energy storage systems.

## Figures and Tables

**Table 1:** Overview of experimental conditions and performance parameters of previous works on acid–base flow batteries (ABFBs) (chronological order).

Authors, Year (Source)	Battery Composition	Membranes	Charge/Discharge Conditions	Performance *
Emrén and Holmström, 1983 [26]	7-triplet stack, copper electrodes. 0.85 M NaCl (all compartments)	Self-made BPM (modified polysulphones), CEM/AEM: not specified. Membrane active area: 7 cm^2^	Charge: 1.4–56 A/m^2^ (for 2 h) Discharge: constant load	Voltage 1.8 V at 1.4 A/m^2^; EE 0.1%
Pretz and Staude, 1998 [27]	20-triplet stack, platinized Ti electrodes. Electrolyte composition: 0.1, 0.5, or 1.0 M NaCl-HCl-NaOH	BPM: Stantech, or self-made by casting or gluing. CEM/AEM: self-made from polysulphones, Thomapor MC3470/MA3475, Tokuyama CMS/ACS	Only discharge: 0–50 A/m^2^	Power density: 3.63 W/m^2^ (10.9 W/m^2^ triplet) with 0.5 M HCl-NaOH; EE 22%
Zholkovskij et al., 1998 [28]	1-triplet stack + extra salt comp., platinized Ti electrodes. Batchwise operation (no flow). 0.03 M HCl-NaOH (acid/base comp.)	BPM: Stantech; CEM/AEM: Selemion CMV/AMV. Membrane active area: 28 cm^2^	Charge: 3.57 A/m^2^ for 50 min, 35.7 A/m^2^ for 5 min, or 357 A/m^2^ for 0.5 min. Discharge: 0.4 mA/m^2^ for 30 h (slow discharge), 1.7 A/m^2^ for 10 min (fast discharge)	At slow discharge (30 h): specific capacity: 0.3 Ah/kg; energy density: 0.1 Wh/kg; max power density: 0.005 W/kg product. At fast discharge (10 min): specific capacity: 0.15 Ah/kg; energy density: 3 × 10^−2^ Wh/kg; max power density: 0.5 W/kg product. Efficiency: 45–61%
Kim et al., 2016 [29]	1-triplet stack, carbon-felt electrodes. Electrolyte composition: 0.1 M NaCl, 0.1, 0.2, …, 0.6, or 0.7 M HCl-NaOH. Electrode rinse solution: 0.01 M FeSO_4_/Fe_2_(SO_4_)_3_, 0.01 M Na_2_SO_4._	BPM: Tokuyama BP-1; CEM/AEM: Tokuyama CMS, CMX/AM-1	13 cycles with 0.5 M HCl-NaOH system at 2.9 A/m^2^. Cycle voltage range: 1.25–0.40 V.	Max power density 2.9 W/m^2^ (11.6 W/m^2^ per single-cell stack) with 0.6 M HCl-NaOH. Cyclability tests with 0.5 M HCl-NaOH: CE 98.3%, VE 76.0%, EE 77.3% from 2nd to 9th cycle on average. Average charge capacity 1.12 Ah/L, average discharge capacity 1.11 Ah/L. Rapid decline in charge/discharge capacities after 9th cycle.
Van Egmond et al., 2018 [25]	1-triplet stack, Ir/Ru-coated Ti electrodes. Electrolyte composition: 0.214 M NaCl (salt comp.), 1 M HCl + 0.5 M NaCl (acid comp.), 1 M NaOH + 0.5 M NaCl (base comp.). Electrode rinse solution: 0.5 M Na_2_SO_4_.	BPM: Fumasep FBM. CEM: Nafion N117. AEM: Fumasep FAB-PK-30 (proton blocking). Membrane active area: 100 cm^2^.	9 charge/discharge cycles. Voltage range: 0–0.83 V. Charge: 50–150 A/m^2^; discharge: 5–15 A/m^2^. Discharge time: 7 h at 5 A/m^2^, and 5 h at 15 A/m^2^	Power density up to 3.7 W/m^2^. Energy density 2.9 Wh/L
Xia et al., 2018 [30]	1-triplet stack + extra salt comp., platinized Ti mesh electrodes. Electrolyte composition: 0.5 M NaCl (salt comp), 0.25, 0.5, 0.75, or 1.0 M HCl-NaOH (acid/base comp.). Electrode rinse solution: 0.25 M Na_2_SO_4_.	BPM: Fumasep FBM; CEM/AEM: Fumasep FKB/FAB. Membrane active area: 25 cm^2^.	20 cycles with 0.75 M HCl-NaOH system at 400 A/m^2^ charge/discharge; 20 min charge, 20 min discharge per cycle	OCV 0.775 V; mean voltage over the BPM: 0.87 V at charge, 0.63 V at discharge (BPM VE 72%)
Xia et al., 2020 [31]	20-triplet stack. Electrolyte composition: 0.5 M NaCl (salt comp), 0.5 or 1.0 M HCl-NaOH (acid/base comp). Electrode rinse solution: 0.25 M Na_2_SO_4_	BPM: Fumasep FBM; CEM/AEM: Fumasep FKB/FAB. Membrane active area: 100 cm^2^.	90 A/m^2^ charge and discharge, both 5 min.	~15 W/m^2^ excluding electrode losses, for 20- triplet stack with 1 M HCl-NaOH at 100 A/m^2^.
Zaffora et al., 2020 [36]	5–38-triplet stack. Ti/mixed-metal oxide electrodes. Electrolyte composition: 0.25 M NaCl (salt comp.), 0.2, 0.6 or 1.0 M HCl-NaOH (acid/base comp). Electrode rinse solution: 0.25 M Na_2_SO_4_ or 0.5 M FaCl_2_/FeCl_3_.	BPM: Fumasep FBM; CEM/AEM: Fumasep FKB/FAB. Membrane active area: 100 cm^2^.	Only discharge with single pass, up to 100 A/m^2^.	~17 W/m^2^ for 10-triplet stack with 1 M HCl-NaOH at 100 A/m^2^. Estimated energy density of 10.3 kWh/m^3^ acid for complete discharge.

* Power density values are normalized per m^2^ of total membrane area (to be multiplied by a factor 3 to obtain a power density per m^2^ of BPM, or triplet). BPM, bipolar membrane; CEM, cation-exchange membrane; AEM, anion-exchange membrane.

**Table 2:** Overview of main input parameters used in the sensitivity analysis. Adapted from Reference [35].

Geometrical Parameters of the Stack				
	**Units**	**Value**		
Spacer length, *L*	cm	50		
Spacer width, *b*	cm	50		
Spacer thickness	μm	475		
**Membrane Properties**				
	**Units**	**AEM**	**CEM**	**BPM**
Thickness	µm	130	130	190
Areal resistance	Ω cm^2^	4.0	3.5	5.0
H^+^ diffusivity ^a^	m^2^/s	2.0 × 10^−11^	0.7 × 10^−11^	-
Na^+^ diffusivity	m^2^/s	1.6 × 10^−11^	0.5 × 10^−11^	-
Cl^−^ diffusivity	m^2^/s	1.7 × 10^−11^	0.6 × 10^−11^	-
OH^−^ diffusivity	m^2^/s	1.9 × 10^−11^	0.6 × 10^−11^	-
Fixed charge density	mol/m^3^	5000	5000	-
**Feed Conditions in the First Stage**				
**Feed Composition**	**Units**	**Charge phase (0% SOC)**	**Discharge (100% SOC)**	
HCl in acid compartment	mol/m^3^	50	1000	
NaCl in acid compartment	mol/m^3^	250		
HCl in salt solution compartment	mol/m^3^	10		
NaCl in salt solution compartment	mol/m^3^	1000		
NaOH in base compartment	mol/m^3^	50		
NaCl in base compartment	mol/m^3^	250		
Fluid flow velocity	cm/s	1.0	1.0	
**Electrode Compartments and Triplets**				
	**Units**	**Value**		
Blank resistance ^b^	Ω cm^2^	12		
Number of triplets (repeating units) per stack, *N*	-	10		

^a^ Ion diffusivities estimated from experimental measurements with NaCl solutions and a two-chamber diffusion cell, assuming ion diffusivities inversely proportional to hydrated radius (Stokes–Einstein equation); ^b^ Blank resistance obtained experimentally by using FeCl_2_/FeCl_3_ as electrode rinse solution. SOC, state of charge.

**Table 3:** Main results predicted by the simulation of the pilot plant. The current density was fixed at 100 A/m^2^ for both charge and discharge.

Quantity	Units	Value
Average external voltage in charge ^1^	V	6.7
Average external voltage in discharge ^1^	V	4.3
Average Gross Power Density in charge	W/m^2^	32.0
Average Gross Power Density in discharge	W/m^2^	20.6
Gross Energy Density in discharge	kWh/m^3^_acid_	18.0
Current Efficiency	-	86.8%
Voltage Efficiency	-	64.4%
Round Trip Efficiency	-	55.9%

^1^ Average voltage over seven triplets.

**Table 4:** Capital expenditure (CAPEX) of current pilot-scale plant (1 kW/7 kWh), and cost estimation for First-of-a-Kind (FOAK) commercial unit (100 kW/700 kWh) in 2025.

CAPEX (Materials)	Demonstration Pilot (2020) 1 kW/7 kWh		FOAK Commercial Unit (2025) 100 kW/700 kWh	
Power subsystem (membrane, spacers, electrodes)	€85,000	€85,000/kW	€152,000	€1520/kW
Energy subsystem (storage tanks, electrolyte)	€13,000	€1900/kWh	€35,000	€50/kWh
Periphery (battery management systems, sensors)	€39,000	€39,000/unit	€22,000	€22,000/unit
System (total)	€137,000	€19,600/kWh	€328,000	€470/kWh
